# After the chemotherapy: potential mechanisms for chemotherapy-induced delayed skeletal muscle dysfunction in survivors of acute lymphoblastic leukaemia in childhood

**DOI:** 10.3389/fphar.2013.00049

**Published:** 2013-04-19

**Authors:** Celena Scheede-Bergdahl, R. Thomas Jagoe

**Affiliations:** ^1^Cancer Nutrition Rehabilitation Program, Department of Oncology, McGill UniversityMontreal, QC, Canada; ^2^The Lady Davis Institute for Medical Research, Segal Cancer Centre, Jewish General HospitalMontreal, QC, Canada

**Keywords:** skeletal muscle, chemotherapy, muscle dysfunction, muscle satellite cells, motor neurons, muscle mitochondria, cancer survivors, acute lymphoblastic leukemia

## Abstract

There is evidence that survivors of childhood cancers, such as acute lymphoblastic leukemia (ALL), have increased rates of long-term skeletal muscle dysfunction. This places them at higher risk of physical restriction and functional impairment as well as potentially contributing to observed increases in cardiovascular disease and insulin resistance in later life. The mechanisms underlying these changes in skeletal muscle are unknown but chemotherapy drugs used in treatment for ALL are strongly implicated. Normal skeletal muscle growth, development, and function are dependent on correctly functioning muscle satellite cells, muscle motor neurons, and muscle mitochondria. Each of these key components is potentially susceptible to damage by chemotherapy in childhood, particularly prolonged courses including repeated administration of combination chemotherapy. If this chemotherapy-induced damage is not fully reversible, impairment of satellite cells, muscle motor innervation, and mitochondria could, either singly or together, lead to the emergence of delayed or persistent skeletal muscle dysfunction many years later. The known effects of individual drugs used in the treatment of ALL are outlined and the need for specific targeted studies to investigate the mechanisms underlying persistent muscle dysfunction in survivors of ALL and other childhood cancers is highlighted.

## INTRODUCTION

Advances in the diagnosis and treatments for pediatric cancers over the last 40 years have led to striking improvements in survival. The use of modern multimodal treatment for one of the commonest childhood cancers, acute lymphoblastic leukemia (ALL), has elevated 5-year survival rates from below 5% in the 1960s (Zuelzer, 1964) to 91% in 2007 (Siegel et al., 2012). Indeed the overall 5-year survival rate for all childhood malignancies is now 83% (Siegel et al., 2012). Although a high proportion of those children treated for cancer will survive their disease and enter adulthood (Oeffinger, 2003), it is becoming increasingly evident that this population is at higher risk of developing health-related problems in adult life (Oeffinger et al., 2006). Indeed in children who were treated for cancer in the 1970s and 1980s and studied on average 17.5 years later, there was a relative risk of 3.3 for developing any chronic medical condition, and 8.2 relative risk of a severe or life-threatening condition as compared with their siblings who did not require treatment for cancer (Oeffinger et al., 2006). Thus the obvious survival benefits of modern treatment methods, must be balanced against compelling evidence that those successfully treated for cancer in childhood have a much higher risk of impaired health, even many years after completion of treatment (Oeffinger et al., 2006). The mechanisms underlying these longer term health effects are largely uncertain and may vary with different types of treatment and possibly between different individual patients depending on their age or stage of development and comorbidities. In addition, tissues or organs have different patterns of growth and capacities for self-renewal, which may make them more or less susceptible to manifest delayed dysfunction or damage long after completion of anti-cancer therapies. In this article we will review evidence that demonstrates the likely long-term impact of chemotherapeutic agents commonly used to treat ALL, on skeletal muscle function. We will outline three distinct, hypothetical but muscle-specific molecular mechanisms, that would explain the observed delayed, persistent, and subacute presentation of skeletal muscle dysfunction. In addition we will discuss the potential of some of the key chemotherapy drugs commonly used in the treatment of childhood ALL, to cause delayed effects on skeletal muscle. Finally we will highlight how loss of skeletal muscle can affect both functional capacity and metabolic health in cancer survivors, and make the case for the need for carefully designed and specifically targeted studies. Such studies will help better understand the global long-term effects of anti-cancer treatments on skeletal muscle, and facilitate the development of safe and effective counter-measures.

## SURVIVORS OF CHILDHOOD CANCER HAVE INCREASED LEVELS OF MUSCLE DYSFUNCTION AND WEAKNESS

Studies of survivors of childhood cancer, including a majority who are patients treated for ALL, confirm that these individuals have significantly increased rates of physical performance limitation and frequently report restrictions in participation in activities needed for day-to-day functions (Ness et al., 2005), many years after the end of treatment. These findings are also reflected in results of exercise testing which demonstrate reduced maximal exercise capacity as assessed using cycle ergometry to measure VO_2_ peak and maximum work load (Talvensaari et al., 1995; van Brussel et al., 2006; Ness et al., 2007; Järvelä et al., 2010) as well as other timed exercise performance tests (Ness et al., 2007). Of course reductions in maximal exercise capacity may result from a number of factors including obesity (Oeffinger et al., 2003) and deconditioning secondary to lower habitual exercise levels (Florin et al., 2007; Warner, 2008), both of which are more common, particularly in female survivors of childhood ALL. However, there is extensive evidence that points more specifically to skeletal muscle dysfunction in long-term survivors of childhood cancers several years after the end of anti-cancer therapy. This includes data showing reduced fat-free mass measured by skinfold method (Talvensaari et al., 1995) and reduced skeletal muscle mass measured by dual-energy X-ray absorptiometry (Ness et al., 2007), in survivors of childhood ALL. Also functional skeletal muscle testing has revealed that long-term survivors of ALL have poorer muscle endurance, assessed using sit-up and push-up maneuvers (Hovi et al., 1993), and reduced limb muscle strength (Hovi et al., 1993; Talvensaari et al., 1995; van Brussel et al., 2006; Ness et al., 2007). In addition more integrated testing of motor proficiency including strength, balance, and agility were also lower in children after treatment for ALL (Harten et al., 1984; Wright et al., 1998). Furthermore the deficits in exercise capacity and muscle function are not simply secondary to impaired heart function, or because of inactivity and deconditioning, as they are demonstrated even in the absence of any cardiac dysfunction in cancer survivors (Järvelä et al., 2010), and even when comparing cancer survivors with age and sex-matched controls with similar low habitual physical activity levels (Järvelä et al., 2010).

## PATTERNS OF SKELETAL MUSCLE DEVELOPMENT RELEVANT TO LONG-TERM CHEMOTHERAPY-INDUCED DAMAGE

Given the evidence suggesting measurable impairments in skeletal muscle function in long-term survivors of ALL, and taking into account the age and developmental immaturity of the typical recipients, a number of mechanisms can be proposed which have specific relevance to muscle tissue. In particular the proposed mechanisms would lead to defects in muscle function that would persist or emerge many months or years after anti-cancer treatments have been completed.

### IMPAIRMENT OF MUSCLE SATELLITE CELLS

During neonatal development there are rapid increases in both muscle protein synthesis and numbers of myonuclei to sustain muscle growth and early functional development (Davis and Fiorotto, 2009). New muscle nuclei originate from activated, proliferating muscle satellite cells that make up a high proportion of total muscle nuclei at birth. Thereafter, data from rats and pigs shows the proportion of muscle satellite cells declines steadily until sexual maturity. In human muscle the total number of muscle fibers appears stable from childhood to middle age (Lexell et al., 1992) but dramatic increases in fiber size (hypertrophy) leads to a doubling of muscle cross-sectional area from childhood to early adult life (Lexell et al., 1992). Muscle satellite cells are essential for this hypertrophy in early development as demonstrated by Pax7- (a muscle satellite cell marker) deficient mice, which have normal muscle fiber number, but severely impaired growth and regenerative capacity after injury (Kuang et al., 2006). In addition maintenance of the muscle satellite cell population requires appropriate functional signals: thus disuse or unloading of limb muscles during development leads to accelerated loss of muscle satellite cells (Kawano et al., 2008).

After puberty, muscle satellite cells are much less active but can be identified in sub-sarcolemmal “niches” (Bentzinger et al., 2012). Though quiescent, these cells retain the ability to be activated in response to a variety of stimuli, including muscle damage, and re-enter the cell cycle and proliferate. The fate of the progeny of this proliferation of activated muscle satellite cells is dichotomous: a proportion fuse with and repair existing myofibers, or in some situations form new myofibers. However, in addition, and equally importantly, some progeny do not progress through muscle differentiation but return to the quiescent state in the niche and thus replenish the pool of muscle satellite cells available for muscle repair functions in the future.

The median age of presentation with ALL is 4 years (Kulkarni et al., 2011), which coincides with the period when muscle satellite cells are still highly active, proliferating, and contributing to muscle growth. Activated satellite cells appear to be more susceptible than quiescent satellite cells to cytotoxic conditions including oxidative stress (Pallafacchina et al., 2010). Thus exposure of activated muscle satellite cells to DNA damaging agents and chemotherapy-induced oxidative stress (Chen et al., 2007) in childhood, may impair the satellite cells’ ability to proliferate and differentiate, or to replenish the quiescent cell population. Furthermore, other effects of treatment during childhood such as enforced inactivity and possible prolonged impairment of motor neuron function, may have additional important roles in depleting the number of satellite cells in muscle. Thus one plausible mechanism for chemotherapy-related muscle dysfunction is that chemotherapy treatment leads to early depletion or accelerated senescence of muscle satellite cells. Any immediate impact of this type of reduction in the net muscle regenerative capacity would be very difficult to detect; but failure to repair and regenerate normally after exercise-related muscle injury over an extended period, would lead to an incremental loss of muscle fibers and a premature decrease in muscle mass.

### IMPAIRED ACTIVITY OF MOTOR NEURONS OR DEPLETION OF MUSCLE MOTOR UNITS

Each motor neuron typically innervates several muscle fibers within a given muscle, collectively known as a motor unit. The nerve conduction velocity and the frequency and pattern of depolarization, as well as the density of innervation of muscle fibers, all play a role in normal skeletal muscle growth, function, and adaptation throughout life (Duchateau et al., 2006). During childhood, acquisition of gross and fine motor skills involves maturation of nerve fiber tracts in the central nervous system through childhood and adolescence, which in turn facilitate development of motor and speech functions (Paus et al., 1999). In the periphery too, nerve fiber conduction patterns mature throughout early childhood, allowing both motor and sensory nerve conduction velocities to increase to adult speeds (García et al., 2000). Neurological deficits are frequently documented in children undergoing treatment for ALL and may be due to direct or indirect effects of the disease or treatments including drugs such as vincristine (see below) and cranial irradiation (Vainionpää, 1993). Peripheral neuropathy and impairment in fine and gross motor skills, including walking, are reported in up to 30% of patients after 2–3 years of therapy (Vainionpää, 1993). While defects such as peripheral neuropathy may be transient and recover over time after completion of treatment, subacute sequelae of the impairments in nerve function and development during childhood, may lead to other secondary effects. Thus children diagnosed with ALL at a young age are at a higher risk for developing restricted joint range of motion, and other chronic changes such as contractures years after completing treatment (Wright et al., 1998). In addition cranial radiotherapy for central nervous system disease, may indirectly impede muscle development and growth through damaging effects on the development of central neural tracts, and via impacts on the hypothalamic-pituitary axis leading to deficiencies in hormones, such as growth hormone, that are important for muscle anabolism (Skinner, 2012a).

During normal aging, and in some primary muscle or neurological diseases, the density of innervation of muscle fibers is progressively reduced through cycles of denervation and incomplete re-innervation (McComas et al., 1993). Eventually the regenerative capacity of motor neuron terminal axons is exceeded leading to loss of motor units, accumulation of severely atrophied fibers, and finally loss of muscle fibers (Deschenes, 2004). This heralds an inexorable loss of muscle mass, strength, and functional capacity. Several anti-cancer treatments, including chemotherapies used in ALL (see below), have the potential to cause motor neuropathies (Balayssac et al., 2011). Given this potential for chemotherapy-induced neural damage, it is also possible that after completing treatment for ALL, muscle fibers are less densely innervated which leaves them more susceptible to accelerated denervation of aging. This in turn would leave affected patients more likely to develop muscle weakness, wasting, and physical frailty earlier or more rapidly than their peers. Thus another mechanistic hypothesis for long-term muscle dysfunction after treatment for ALL would be that sub-clinical, generalized, chemotherapy-related damage to motor neurons or neuromuscular junctions, promotes progressive denervation-related muscle loss.

### IMPAIRED MUSCLE MITOCHONDRIAL FUNCTION

Mitochondria in muscle are essential sources of energy (ATP) for performance of external contractile work, but are also involved in any other aspects of muscle tissue homeostasis including regulating atrophy pathways and apoptosis (Marzetti et al., 2010). Normal mitochondrial biogenesis and functioning is dependent on tightly regulated expression of genes encoded on both nuclear and mitochondrial (mt) DNA. mtDNA is susceptible to damage by oxidative stress (Doria et al., 2012) and mutates at higher rates than nuclear DNA (Barja and Herrero, 2000). Deletion mutations in mtDNA can affect expression of mitochondrial proteins including components of the electron transport chain, resulting in impaired mitochondrial function, and have been observed in many different diseases (Lax et al., 2011; Medikayala et al., 2011). To cause functional defects in muscle the proportion of affected mtDNA molecules and mitochondria must reach a certain threshold (Park and Larsson, 2011). mtDNA deletions are propagated through mtDNA replication and mitochondrial biogenesis within muscle and can be amplified in over time such that the threshold causing detectable mitochondrial functional defects is reached several months or years later.

Many anti-cancer treatments including cytotoxic chemotherapies, induce cell death by disrupting normal nuclear DNA repair and replication. In mononucleated cells this leads to cessation of proliferation and cell death (along with associated loss of any cellular mitochondria and mtDNA), which is the basis for the anti-cancer activity of such drugs. However, in post-mitotic multinucleated muscle fibers, damaged nuclear DNA can be cleaved and degraded, without loss of all the other cellular contents, and thus it is possible that acquired mutated mtDNA can persist and accumulate, even if myonuclei containing mutated nuclear DNA are removed. There is evidence that those treated with intensive combined anti-cancer chemotherapy and radiation have increases levels of mutations in muscle mtDNA months or years later (Wardell et al., 2003). However, any functional impact of such mutations might take some years to become evident through mtDNA replication, and progressive amplification of mtDNA mutation load over time. This may be the explanation for findings from a small post-mortem study in humans who died only a few weeks or months after their last treatment. In this study there was evidence of acute cardiac muscle mitochondrial structural and DNA damage, but no evidence of skeletal muscle mitochondrial ultrastructural changes or mitochondrial functional defects (Lebrecht et al., 2005). Thus another hypothesis to explain how chemotherapy in childhood for ALL may lead to delayed skeletal muscle functional impairment, is through progressive amplification and propagation of chemotherapy-induced mtDNA mutations leading to delayed muscle mitochondrial dysfunction.

## CHEMOTHERAPEUTIC AGENTS THAT MAY INDUCE LONG-TERM SKELETAL MUSCLE DYSFUNCTION IN ALL

The fact that the deficits in maintenance of skeletal muscle and function occur or persist many years after successful treatment for childhood cancers such as ALL, strongly suggests that anti-cancer treatments themselves are implicated in causing the skeletal muscle dysfunction described. Direct effects of systemically administered chemotherapeutic agents, which circulate to all tissues in the blood, are prime candidates. Non-tumor tissues, including skeletal muscle, are exposed to a variety of chemotherapy-related toxic effects over the course of treatment with most standard chemotherapy regimes. These include repeated cycles of acute exposure to high levels of oxidative stress (Chen et al., 2007) which, if not controlled by endogenous anti-oxidant defenses, can modify many different macromolecules (nucleic acids, proteins, lipids) and disrupt a wide range of cellular functions. Furthermore many anti-cancer treatments also induce DNA damage through a variety of more direct mechanisms (Jackson and Bartek, 2009). Both these types of cellular toxicity are potentially relevant in initiating changes in muscle tissue in childhood, which lead to the long-term functional deficits described.

Many of the chemotherapy drugs used for treatment of ALL in the 1980s and 1990s remain in use today because they are used in treatment regimes that achieve very good survival rates (Siegel et al., 2012). Induction combination chemotherapy commonly includes treatment with vincristine, corticosteroids, asparaginase and an anthracycline (Pui and Evans, 2006). For all of these drugs, except asparaginase, there is evidence of short-term effects on muscle for each drug individually, as detailed below. However, it should be stressed that the potential synergistic interactions between these and other agents when given concurrently, and the nature of any latent effects on muscle, months or years beyond the period of chemotherapy, are much less clear.

Vincristine is a vinca alkaloid that disrupts microtubules and arrests cell cycle division (Jordan et al., 1985) and is highly active in treatment of ALL. Disruption of microtubular axonal transport is also thought to explain the main side-effects of vincristine treatment, including sensory neuropathy and neuropathic pain, but this drug can also cause motor and autonomic neuropathy (Moore and Pinkerton, 2009; Balayssac et al., 2011). Musculoskeletal abnormalities including neuropathic-induced contractures have been reported in children previously treated for leukemia with vincristine (Ryan and Emami, 1983). The side-effects vary greatly with each individual, and earlier reports suggested the neuropathy was largely reversible on completion of treatment (Rosenthal and Kaufman, 1974). However, more recent reports have documented mobility and musculoskeletal impairments in long-term survivors of ALL, and identified that higher cumulative doses of vincristine increase the risk for such persisting neuromuscular defects (Ness et al., 2012).

Glucocorticoids, including drugs such as dexamethasone and prednisone, are frequently used for their anti-emetic properties around the time of chemotherapy (Jordan et al., 2011), but higher and more prolonged courses are used for their proven specific anti-neoplastic role in hematological malignancies such as ALL (Pui and Evans, 2006). During administration of glucocorticoids, especially prolonged courses or higher doses, skeletal muscle atrophy can be expected in 40–60% of patients (Batchelor et al., 1997; Lee et al., 2006). Steroid myopathy is more pronounced in proximal muscles and fast twitch (type II) fibers (Schakman et al., 2008). Muscle wasting is initially due to activation of muscle proteolysis (Baehr et al., 2011) and later due to insulin resistance and impaired muscle protein synthesis and anabolism (Menconi et al., 2007). Also recent data in myotube culture shows that glucocorticoids promote muscle-specific gene expression (Pansters et al., 2012) and this property could conceivably disrupt the ability of a proportion of muscle satellite cells to return to the undifferentiated quiescent state after initial proliferation. Finally corticosteroids increase appetite and this and other metabolic changes frequently results in obesity in children undergoing treatment for ALL, though in many children percentage body fat and body composition improves when treatment is completed (van der Sluis et al., 2002). While it is not clear whether high dose glucocorticoids contribute to long-term muscle dysfunction, in children treated for ALL, there is evidence from adults that prolonged corticosteroid use can induce muscle mitochondrial dysfunction and increased oxidative damage to both nuclear and mtDNA (Mitsui et al., 2002).

Anthracyclines, such as doxorubicin, bind DNA avidly and preferentially accumulate in nuclei and mitochondrial. The DNA-bound drug interferes with nuclear DNA transcription and replication, and thus inhibits cell growth and division (Gewirtz, 1999). These highly effective anti-cancer agents are still routinely used in ALL, particularly in children with less responsive disease; but anthracyclines are also known to cause potentially life-threatening cardiomyopathy (Octavia et al., 2012), and the frequency and severity of this cardiotoxicity increases with higher cumulative doses (Ewer and Ewer, 2010). Cardiac muscle toxicity of anthracyclines is mediated through increased production of reactive oxygen species (ROS) by cardiac muscle mitochondria (Octavia et al., 2012), and increased intracellular calcium levels which may promote ROS production and impair contractile function (Octavia et al., 2012). Direct mtDNA binding by anthracyclines and increased mitochondrial ROS production may both contribute to observed increased mtDNA damage which accumulates with repeated treatments (Serrano et al., 1999). Different tissues accumulate anthracycline-induced mtDNA damage at varying rates, but in rats increased mtDNA mutations, defective mitochondrial respiratory enzyme activity and increased ROS production are observed in cardiac (Lebrecht et al., 2003) and renal (Lebrecht et al., 2004) tissues, 6–7 months after the end of treatment. Early studies suggested skeletal muscle was not a target for acute anthracycline-induced damage (Ito et al., 1990) or mitochondrial dysfunction or ultrastructural changes (Lebrecht et al., 2003). However, in contrast, there are recent data from tissue culture and rodent studies which demonstrate that acute administration of doxorubicin, induces skeletal muscle mitochondrial ROS, increases proteolysis and leads to muscle weakness (Gilliam et al., 2009, 2012; Gilliam and St Clair, 2011). As a result, though acute toxicity has been demonstrated experimentally in skeletal muscle, it is still uncertain whether anthracyclines used in treatment of childhood ALL also induce persistent long-term skeletal muscle dysfunction, either through mtDNA mutations and defective respiration, or other mechanisms such as altered calcium handling (van Norren et al., 2009).

Other anti-cancer chemotherapy drugs have also been associated with skeletal muscle dysfunction but for many of them the evidence of a direct effect on skeletal muscle is currently less persuasive than for the agents discussed above. For example retrospective analyses have linked the use of methotrexate (Ness et al., 2012) and asparaginase (Hovi et al., 1993) with the development of more severe persistent musculoskeletal limitations in survivors of ALL. Other reports have implicated cytosine arabinoside as a causative agent in acute severe muscle damage after treatment for ALL (Morales-Polanco et al., 1997). In addition newer targeted drugs such as the tyrosine-kinase inhibitor, Imatinib, are being investigated as potentially useful treatments for maintenance therapy in ALL (Rives et al., 2011). However, there is not yet enough clinical follow-up data to know whether these newer drugs also have deleterious long-term impacts on skeletal muscle or other tissues. Thus, it remains possible that the specific agents discussed above, and others used in the treatment of ALL have a synergistic impact on muscle, and it is self-evident that distinguishing the precise role of each drug in causing long-term muscle dysfunction is extremely challenging. Nevertheless, from a therapeutic standpoint the more important question is not whether each individual drug does or does not damage skeletal muscle, but (a) what is the nature of the impact of the combination treatments being employed for ALL? and (b) how can any deleterious effects on muscle be avoided or minimized?. If it is true that chemotherapy given in childhood causes long-term muscle dysfunction, and given that current treatment regimes lead to very high cure rates for ALL, we can anticipate that these agents will continue to be used, and thus will likely cause long-term muscle dysfunction in the growing number of adults who will survive current treatment of ALL.

## THE BROADER IMPLICATIONS OF LONG-TERM CHEMOTHERAPY-INDUCED SKELETAL MUSCLE DYSFUNCTION

As discussed above there is compelling evidence that, as a group, children who have successfully completed treatment for ALL (or indeed other childhood cancers), have diminished health status as adults. Furthermore, a higher proportion of these individuals will also have reduced muscle strength and function. Although the mechanisms underlying the observed muscle dysfunction are not clear, it is likely that chemotherapy treatments have an important direct role. The growing awareness of the late effects of cancer and cancer treatments has stimulated calls to establish long-term surveillance for the survivors of pediatric cancers (Aslett et al., 2007; Oeffinger et al., 2010; Prasad et al., 2010; Skinner, 2012b). If successful such programs will allow more systematic identification and characterization of affected individuals, and will hopefully facilitate studies to investigate the hypotheses proposed here and to initiate potential intervention studies.

Long-term chemotherapy-induced toxicity in skeletal muscle is relatively poorly studied, despite the importance of this tissue in determining mobility and physical function. Furthermore, skeletal muscle serves metabolic as well as contractile functions, including being a principal site of lipid oxidation and glucose uptake, and thus a major determinant of insulin sensitivity. Hence chemotherapy-induced loss of skeletal muscle mass, or other types of skeletal muscle dysfunction, may make a direct or indirect contribution to the increased morbidity secondary to chronic medical conditions (Oeffinger et al., 2006) such as obesity (Oeffinger et al., 2003; Curry et al., 2006), cardiovascular disease (Nagarajan et al., 2011), and metabolic syndrome (Talvensaari et al., 1996), which are observed in pediatric cancer survivors. Moreover some, or all, of the chemotherapy-induced effects on muscle tissue discussed above in children, may also be relevant to the far larger number of adults undergoing chemotherapy treatment. If this is true, it follows that functional and metabolic limitations due to skeletal muscle dysfunction are also leading to increased morbidity and mortality in long-term survivors of adult cancers; but that these chemotherapy-related effects are currently being misattributed to aging or other chronic degenerative diseases.

### FUTURE DIRECTIONS

For now the mechanisms by which skeletal muscle dysfunction occur in survivors of childhood cancer (or in adults) remains an open question. But this question can only be answered by specific targeted studies. We have outlined three cellular mechanisms for long-term muscle dysfunction involving impairment of muscle satellite cells, muscle denervation, and muscle mitochondrial dysfunction. These mechanisms, have specific relevance to muscle tissue and are all plausible sequelae of intensive chemotherapy treatment, particularly in childhood. New studies to test these hypotheses in robust experimental models and, most importantly, in cancer survivors, are now needed. Experimental models, including animal models, can help verify whether any of the mechanistic hypotheses set out above can be confirmed. However, animal models can only go so far in replicating the impact of 10–15 years of survival after cancer therapy in humans. Thus carefully designed clinical studies, incorporating muscle biopsies of adult survivors of childhood ALL will be required for final confirmation. Without such studies, the true cause of the underlying skeletal muscle impairments in these patients will continue to be missed, and this will greatly impede any attempts to devise successful treatment strategies.

## Conflict of Interest Statement

The authors declare that the research was conducted in the absence of any commercial or financial relationships that could be construed as a potential conflict of interest.
